# ^18^F-FDG PET/CT semi-quantitative parameters combined with SCC-Ag in predicting lymph node metastasis in stage I-II cervical cancer

**DOI:** 10.3389/fonc.2024.1278464

**Published:** 2024-06-14

**Authors:** Cheng-Zhi Jiang, Kai Zheng, Yan-Yin Zhang, Jian Yang, Hui Ye, Xiang Peng

**Affiliations:** Department of PET-CT Center, Hunan Cancer Hospital/The Affiliated Cancer Hospital of Xiangya School of Medicine, Central South University, Changsha, Hunan, China

**Keywords:** cervical cancer, positron emission tomography/computed tomography, total lesion glycolysis, lymph node metastasis, squamous cell carcinoma-antigen

## Abstract

**Objective:**

To explore the value of ^18^F-fluordeoxyglucose positron emission tomography/computed tomography (^18^F-FDG PET/CT) semi-quantitative parameters of primary tumor combined with squamous cell carcinoma antigen (SCC-Ag) in predicting lymph node metastasis (LNM) of cervical cancer (FIGO 2018 stage I-II).

**Materials and Methods:**

A total of 65 patients with stage I-II cervical cancer underwent ^18^F-FDG PET/CT were included in our study. Comparing the primary tumor ^18^F-FDG PET/CT semi-quantitative parameters and SCC-Ag between the LNM group and the non-LNM group. Logistic regression and receiver operating characteristic (ROC) were used to analyze the value of ^18^F-FDG PET/CT metabolic parameters and SCC-Ag in predicting LNM.

**Results:**

There were 14 and 51 patients were classified as having LNM and NLNM. The semi-quantitative parameters, including the maximum standardized uptake value (SUVmax), the mean standardized uptake value (SUVmean), the peak standardized uptake value (SUVpeak), the total lesion glycolysis (TLG), the metabolic tumor volume (MTV) of the tumor and SCC-Ag were all significantly higher in LNM than in NLNM (SUVmax, 16.07 ± 7.81 vs 11.19 ± 4.73, SUVmean, 9.16 ± 3.48 vs 6.29 ± 2.52, SUVpeak, 12.70 ± 5.26 vs 7.65 ± 3.26, MTV, 22.77 ± 12.36 vs 7.09 ± 5.21, TLG, 211.01 ± 154.25 vs 43.38 ± 36.17, SCC-Ag, 5.39 ± 4.56 vs 2.13 ± 2.50, all *p*<0.01). Logistic regression analysis showed that TLG was an independent predictor of LNM in stage I-II cervical cancer (OR 1.032, 95% CI 1.013–1.052, *p*<0.01). Moreover, the predictive value of TLG combined with SUVpeak and SCC-Ag increased and the area under the curve increased compared SUVpeak and SCC-Ag.

**Conclusion:**

^18^F-FDG PET/CT semi-quantitative parameters and SCC-Ag have promise for assessing LNM in stage I-II cervical cancer. TLG of primary tumor provides independent and increasing values in predicting LNM in stage I-II cervical cancer.

## Introduction

Cervical cancer is the fourth most common cancer of morbidity and mortality in women, with mortality rates higher in developing countries (1). The presence of lymph node metastasis (LNM) in cervical cancer patients is associated with an increased risk of cancer recurrence and poorer prognosis (2–4). Currently, treatment decisions and prognostic assessments for cervical cancer patients primarily rely on the Federation International of Gynecology and Obstetrics (FIGO) staging system. According to FIGO 2018 for cervical cancer, regardless of tumor size or parametrial infiltration, the presence of LNM is categorized as stage IIIC (1). Locally advanced cervical cancer, including stage IB3-IVA, is typically treated with platinum-based chemotherapy combined with external beam radiotherapy followed by brachytherapy without surgery (5, 6). This classification highlights the significance of LNM in determining disease progression and guiding treatment strategies for cervical cancer patients. Patients with LNM need radiotherapy or chemotherapy after surgery.

In the management of stage I-II cervical cancer, the primary treatment options typically involve an extensive hysterectomy along with bilateral pelvic lymph node resection (1, 7). However, excessive excision can cause a series of LN complications, such as lymphocysts and lower extremity lymphedema, and controversy remains whether pelvic lymph node dissection is necessary (7). This has sparked debates and uncertainties regarding the necessity of pelvic lymph node dissection in stage I-II cervical cancer. Therefore, the detection of preoperative LNM is a crucial issue for patient counseling for developing individualized treatment plans, improving prognosis, and reducing mortality (7, 8).

Magnetic resonance imaging (MRI) and computed tomography (CT) are commonly employed diagnostic tools in the evaluation of cervical cancer. MRI and CT provide valuable information regarding tumor size, local invasion, and LNM. Multi-parameter MRI can improve the diagnostic efficiency of lymph node metastasis (8). However, MRI and CT have limitations when it comes to accurately diagnosis of LNM (9, 10). Recently, ^18^F-fluordeoxyglucose positron emission tomography/computed tomography (^18^F-FDG PET/CT) has emerged as a valuable tool in the diagnosis of cervical cancer LNM and provides quantified metabolic information and anatomical details about cervical lesions and lymph nodes (11, 12). In addition, ^18^F-FDG PET/CT can guide the mapping of cervical cancer radiotherapy targets (13). However, ^18^F-FDG PET/CT also has its limitations. False positive results could occur due to various factors such as inflammation or infection, while false negative results may be seen in small LNM or tumors with low glucose metabolism. The maximum standardized uptake value (SUVmax) has limited value in accurately predicting LNM of cervical cancer (14). The SUVmax represents the highest level of FDG uptake within a lesion, but it does not provide information about the overall metabolic activity or heterogeneity of the lesion. Additionally, the partial volume effect was another limitation that affect the diagnosis of LNM in cervical cancer (14). Therefore, recent research suggests that peak standardized uptake value (SUVpeak), total lesion glycolysis (TLG) and metabolic tumor volume (MTV) have high clinical application values in the evaluation of malignant tumor LNM (14, 15).

In addition, serum tumor markers are gradually being applied in the early diagnosis of cervical cancer. Malignant tumors contain special gene expression products, among which squamous cell carcinoma-antigen (SCC-Ag) is the most common tumor marker for cervical cancer (16). Some scholars have shown that the SCC-Ag levels are correlated with the degree of metastasis and infiltration of cervical cancer, which has certain predictive significance for cervical cancer recurrence (16, 17). In this article, we explored the value of FDG PET/CT metabolic parameters of primary tumor combined with SCC-Ag expression in predicting pelvic LNM in stage I-II cervical cancer patients.

## Materials and methods

### Patients

**Figure 1:**
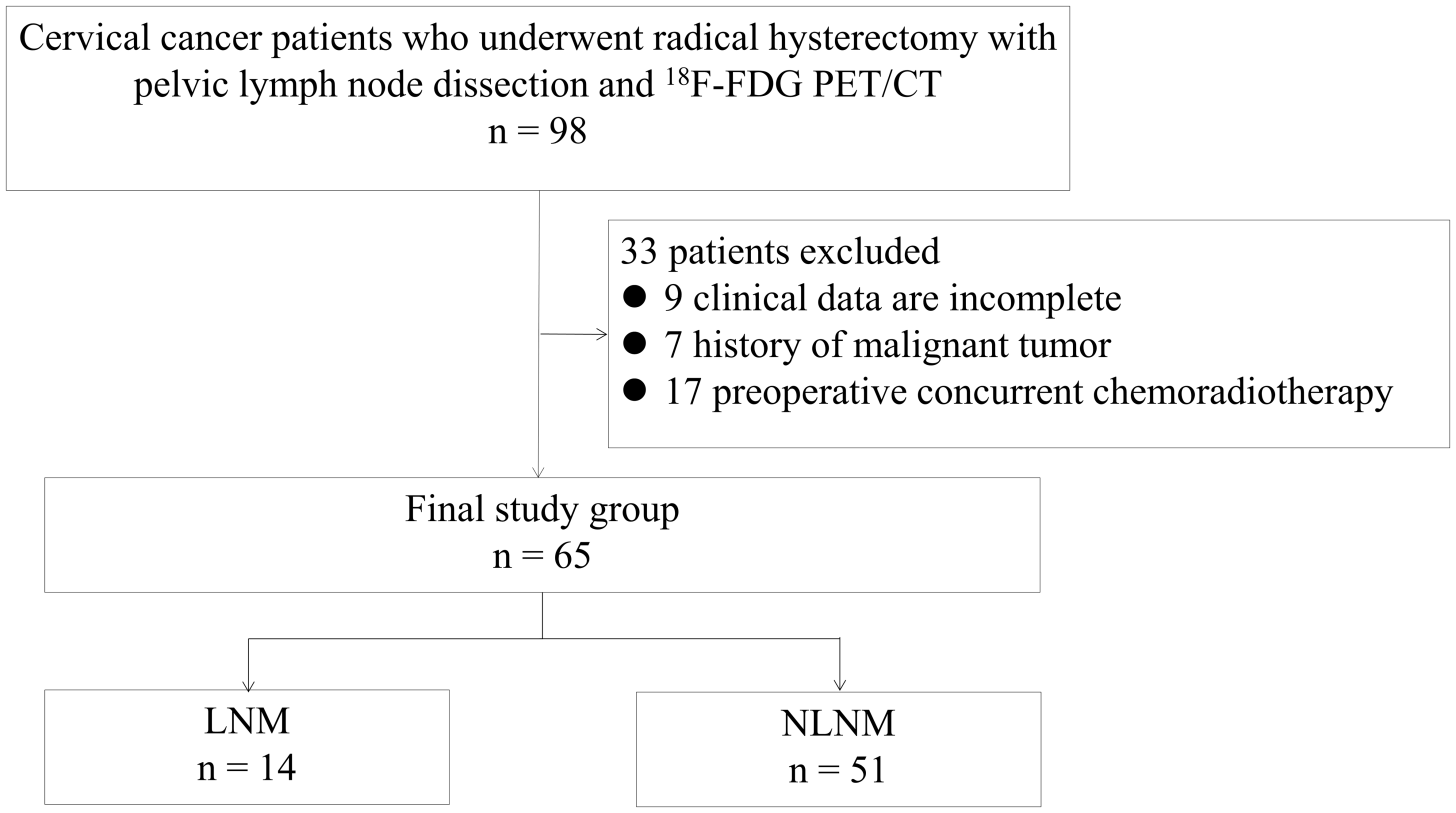
Flowchart for patient enrollment. LNM, lymph node metastasis; NLNM, non-lymph node metastasis.

This retrospective study was conducted with the approval of the Ethics Committee of Hunan Cancer Hospital. Informed consent was obtained from all patients participating in the study, both for imaging procedures and for their data to be used in anonymized analyses. The study included a total of 98 stage I-II cervical cancer patients who underwent ^18^F-FDG PET/CT at a single center between January 2016 and December 2021. Patient inclusion criteria were as follows: (1) biopsy confirmed for cervical cancer, and accepted with radical abdominal hysterectomy and pelvic lymph node cleaning with or without aortic lymph node cleaning, (2) had not received any form of radiotherapy or chemotherapy, (3) did not have other malignant tumors. As a result, 33 patients were excluded from the study based on inclusion criteria: 9 patients clinical data was incomplete, 7 patients had a history of other malignant tumors, and 17 patients underwent preoperative concurrent chemoradiotherapy. Finally, 65 patients were included in our research (**Figure 1**). The clinical data of patients, including age, pathology, SCC-Ag, carcinoembryonic antigen (CEA), and carbohydrate antigen 125 (CA-125), were retrospectively obtained from the medical record center of Hunan Cancer Hospital.

### ^18^F-FDG PET/CT acquisition

All subjects were scanned with a PET/CT scanner (January 2016 to September 2019, Discovery ST, GE, America; October 2019 to December 2021, Discovery MI, GE, America). After 60–90 minutes of intravenous administration of 3.7 MBq/kg ^18^F-FDG (Beijing PET Technology CO.,ltd, Beijing, China), a body noncontrast-enhanced low dose CT (tube voltage of 110 kV, current of 120 mA) was obtained. CT scan was performed from the head to upper-thigh and slice thickness of 3.75 mm. A PET scan was immediately performed after the CT scan in 3D acquisition mode with 6 - 8 bed positions and 2 min per bed position. Later, the CT and PET images were then transferred to an AW4.7 workstation (GE, America), where they were loaded for further analysis by our researchers.

### ^18^F-FDG PET/CT semi-quantitative parameters measurement

PET and CT images were fused at AW 4.7 workstation, and image quality control was performed. ^18^F-FDG PET/CT imaging results were independently assessed by two nuclear medicine specialists (C.Z.J and X.P.) who worked for 5–10 years. When the ^18^F-FDG PET/CT results were inconsistent, another experienced nuclear medicine expert was required to re-evaluate. In the axial fusion image of ^18^F-FDG PET/CT, the plane with clear lesions was selected to delineate the region of interest (ROI) of the primary tumor of cervical cancer, and the SUVmax, the mean standardized uptake value (SUVmean), SUVpeak, TLG and MTV. In order to calculate the MTV, a specific threshold of 40% of SUVmax was applied (18). An SUVmax of the lymph nodes over 2.5 was considered positive lymph nodes and acted as PET/CT LNM.

### Pathological diagnosis

Hematoxylin-eosin (HE) staining was used to stain pathological sections of the patients. Analysis of each patient’s pathology results, including the following contents: the pathological histology type, pathological differentiation grade, cervical stromal invasion depth, LNM, lymphatic vascular space invasion, vaginal metastasis, and parametrial metastasis. Cervical cancer lymph node metastasis includes external iliac, common iliac, obturator, paracral, anterior sacral and paraaortic lymph node metastasis.

### Statistical analysis

In order to analyze the data collected in this study, various statistical methods were employed. The continuous variables were expressed as mean ± standard deviation (SD). According to the nature of the data, continuous variables were compared between groups using Student’s t-test, Mann-Whitney U test, and one-way ANOVA. Classification variables were expressed as a percentage and numbers, and through the Fisher’s exact test. Univariate and multivariate logistic regression analysis were used to analyze the risk factors of cervical cancer LNM and construct the prediction model of LNM. Variables with *p* < 0.05 were included in the model. In addition, the receiver operator characteristic (ROC) curves of different parameters were compared to evaluate the efficacy of different models in predicting LNM. IBM SPSS 25.0 software (IBM Corp., Armonk, NY, USA) was used for statistically analysis. *P* < 0.05 was used to determine the statistical significance of the observation.

## Results

### Patient characteristics

Among the 65 stage I-II cervical cancer patients, 14 patients had lymph node metastasis as the LNM group and 51 patients had no-lymph node metastasis as the NLNM group. The age of the LNM group was 52 ± 12 years, which was not significantly different from that of the NLNM group (55 ± 9 years). The analysis revealed significant differences in cervical stromal invasion depth and parametrial invasion between LNM and NLNM (p<0.05). There were no significant differences in FIGO stage, histologic type, vascular invasion or vaginal stump were found between the LNM and NLNM subjects. The study summarized the clinicopathological characteristics of the participants in **Table 1**.

**Table 1:** Patients’ clinicopathological characteristics.

Clinical feature	LNM (N= 14)	NLNM (N= 51)	*p-value*
Age (years)	52 ± 12	55 ± 9	0.299
FIGO stage, n I II	6 8	30 21	0.287
Differentiation grade, n Well + Moderately differentiated Poorly differentiated	7 7	40 11	0.035
Histologic type, n Squamous carcinoma Adenocarcinoma	10 4	45 6	0.123
Cervical stromal invasion depth, n ≥ 2/3 < 2/3	12 2	23 28	0.007
Vascular invasion, n Yes No	7 7	17 34	0.252
Parametrial invasion, n Yes No	2 12	0 51	0.006
Vaginal stump, n Positive Negative	1 13	2 49	0.611

FIGO, International Federation of Gynecology and Obstetrics; LNM, lymph node metastasis; NLNM, non-lymph node metastasis.

### Comparison of FDG semi-quantitative parameters and tumor markers

Our study evaluated the diagnostic performance of ^18^F-FDG PET/CT in detecting LNM in patients with stage I-II cervical cancer. The results showed that the sensitivity, specificity, accuracy, positive predictive value and negative predictive value were 64.3%, 90.2%, 84.6%, 64.3%, and 90.2%, respectively.

In the LNM group, the SUVmax, SUVmean, and SUVpeak of tumors were obviously higher than that of the NLNM (*p*<0.01). Compared with the NLNM groups, the MTV and TLG of tumors in the LNM group were significantly increased (*p*<0.001). LNM patients revealed a significantly increased CA-125 and SCC-Ag levels compared with NLNM patients (*p*<0.01). Between the NLM and the NLNM group, the CEA levels was no significant difference (**Table 2**).

**Table 2:** FDG semi-quantitative parameters and tumor markers between two groups.

	LNM	NLNM	*p-value*
SUVmax	16.07 ± 7.81	11.19 ± 4.73	0.005
SUVmean	9.16 ± 3.48	6.29 ± 2.52	0.001
SUVpeak	12.70 ± 5.26	7.65 ± 3.26	0.000
TLG	211.01 ± 154.25	43.38 ± 36.17	0.000
MTV	22.77 ± 12.36	7.09 ± 5.21	0.000
CEA	4.16 ± 4.36	2.73 ± 5.98	0.408
CA-125	114.34 ± 236.38	13.49 ± 12.91	0.003
SCC-Ag	5.39 ± 4.56	2.13 ± 2.50	0.001

LNM, lymph node metastasis; NLNM, non-lymph node metastasis; SUVmax, maximum standardized uptake value; SUVmean, mean standardized uptake value; SUVpeak, peak standardized uptake value; TLG, total lesion glycolysis; MTV, metabolic tumor volume; CEA, carcinoembryonic antigen; CA-125, carbohydrate antigen 125; SCC-Ag, squamous cell carcinoma antigen.

### Variables for predicting LNM in stage I-II cervical cancer

Univariate and multivariate logistic regression analyses were further performed to identify the predictive factors for evaluating LNM in stage I-II cervical cancer, including SUVmax, SUVpeak, TLG of tumor, and SCC-Ag. In the univariate analysis, TLG of the tumor (OR: 1.029, 95%CI: 1.005–1.053, *p* = 0.017) was significantly associated with LNM. In the multivariate analysis, TLG of the tumor (OR: 1.032, 95%CI: 1.013–1.052, *p* = 0.001) emerged as a significant and independent predictor of LNM in stage I-II cervical cancer. The result of the analysis are presented in **Table 3**.

**Table 3:** Univariate and multivariate logistic regression analyses in predicting LNM in Cervical Cancer.

Variables	Univariate analysis			Multivariate analysis		
	OR	95%CI	*p-value*	OR	95%CI	*p-value*
SUVmax	0.926	0.632–1.358	0.695			
SUVpeak	1.140	0.643–2.023	0.653			
TLG	1.029	1.005–1.053	0.017	1.032	1.013–1.052	0.001
SCC-Ag	1.078	0.798–1.455	0.624			

SUVmax, maximum standardized uptake value; SUVpeak, peak standardized uptake value; TLG, total lesion glycolysis; SCC-Ag, squamous cell carcinoma antigen.

**Figure 2:**
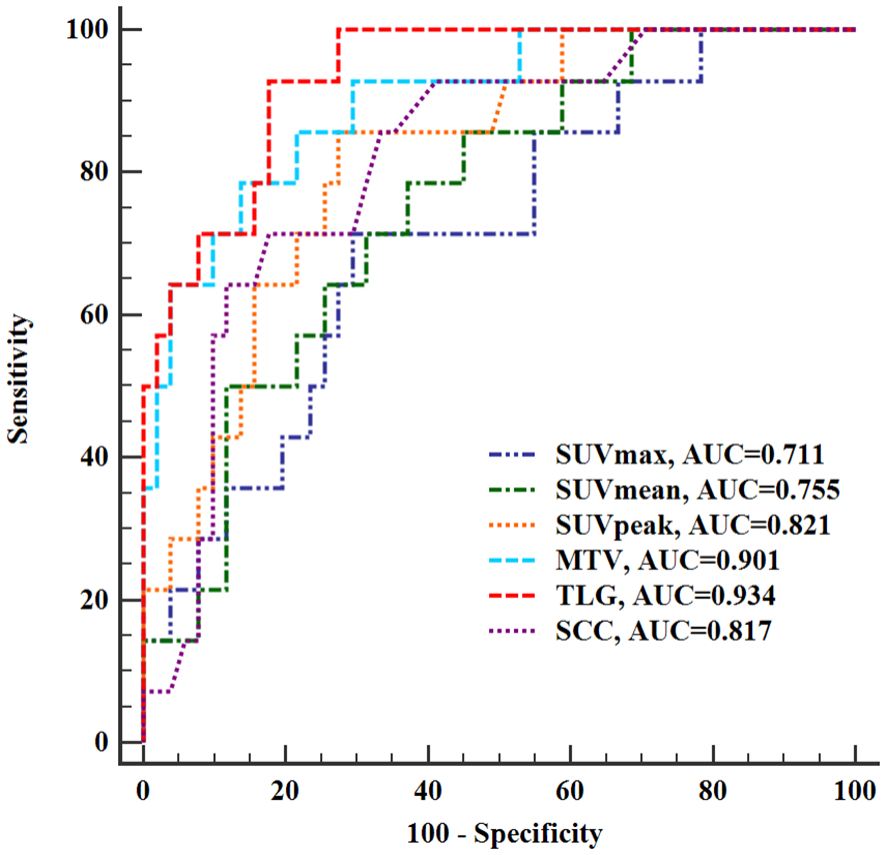
Receiver operating characteristic curves of PET/CT semi-quantitative parameters and SCC-Ag for evaluating lymph node metastasis. SUVmax, maximum standardized uptake value; SUVmean, mean standardized uptake value; SUVpeak, peak standardized uptake value; TLG, total lesion glycolysis; MTV, metabolic tumor volume; SCC-Ag, squamous cell carcinoma antigen; AUC, area under the curve.

ROC analysis showed that the SUVmax (AUC 0.711, sensitivity 71.43%, specificity 70.59%), SUVmean (AUC 0.755, sensitivity 78.57%, specificity 62.75%), SUVpeak (AUC 0.821, sensitivity 85.71%, specificity 72.55%), TLG (AUC 0.934, sensitivity 92.86%, specificity 82.35), MTV (AUC 0.901, sensitivity 78.57%, specificity 86.27%) of the tumor, and SCC-Ag (AUC 0.817, sensitivity 71.43%, specificity 82.35%) had a significant and positive impact on predicting LNM in stage I-II cervical cancer (**Figure 2**). Among the variables analyzed, the TLG of tumor showed the highest diagnostic performance in predicting LNM, and the cut off value of TLG is 72.18.

**Figure 3:**
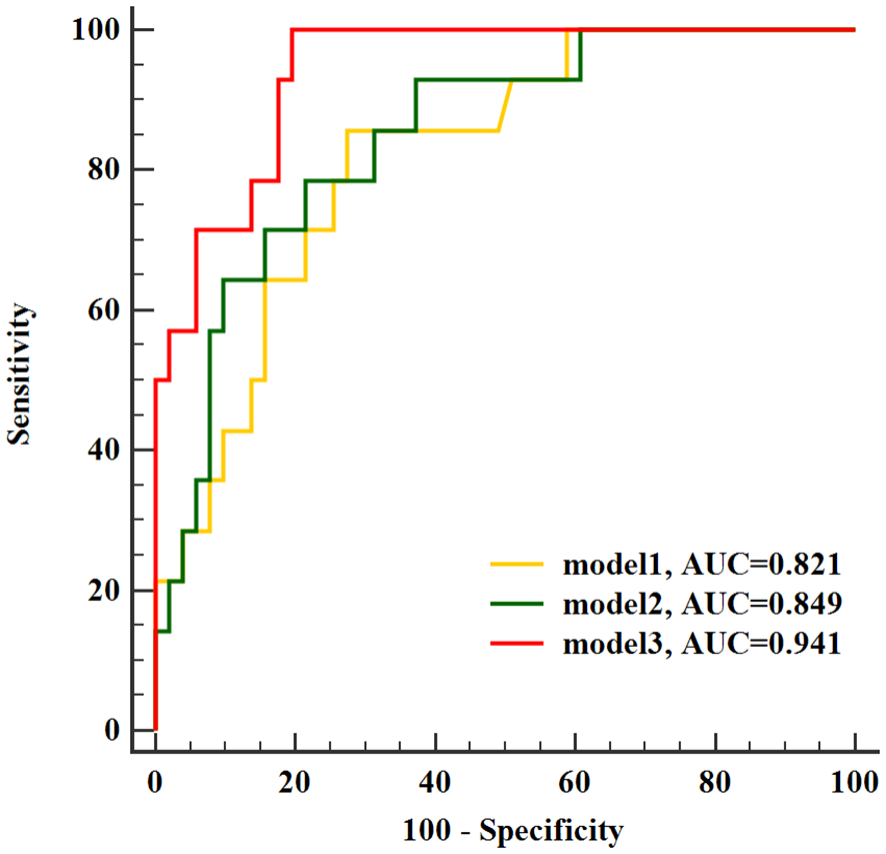
Receiver operating characteristic curves of identified models for evaluated lymph node metastasis. AUC, area under the curve.

In ROC analysis conducted to evaluate predictive models for LNM in stage I-II cervical cancer, Model 1, the SUVpeak of tumor, exhibited the lowest AUC as 0.821 (sensitivity 85.71%, specificity 72.55%). Model 2 combined SUVpeak of the tumor and SCC-Ag (AUC 0.849, sensitivity 78.57%, specificity78.43%), and Model 3 combined SUVpeak of the tumor, SCC-Ag and TLG of the tumor (AUC 0.941, sensitivity 100%, specificity 80.39%). Furthermore, the TLG of tumor provided a significant incremental predictive value for LNM in stage I-II cervical cancer. There was no significant difference in the area under ROC curve between Model 1 and Model 2 (*p* = 0.283). Model 3 further yielded a greater AUC than Model 1and Model 2 (*p* = 0.012 and *p*= 0.045, respectively) (**Figure 3**).

## Discussion

LNM plays a crucial role as an independent and important prognostic factor in cervical cancer (1–4). The presence of lymph node involvement is associated with a higher risk of disease progression, recurrence, and poorer overall survival outcomes. The treatment decision for cervical cancer is mainly based on FIGO staging. In the management of stage I-II cervical cancer, surgical intervention is considered the primary treatment approach. The standard treatment methods for stage I-II cervical cancer include radical hysterectomy and pelvic lymph node resection (19). Therefore, preoperative prediction and evaluation of LNM have important value for clinical intervention in cervical cancer. In recent years, several advanced imaging techniques, such as CT, MRI, and ^18^F-FDG PET/CT, have shown promise in the detection of LNM in cervical cancer. A lymph node diameter greater than 10 mm is one of the important criteria for routine imaging diagnosis of LNM, but diagnostic accuracy for detecting LNM is still limited in clinical practice (2, 9, 10). Assessing LNM using CT and MRI poses a significant challenge in clinical practice. While CT and MRI scans provide valuable information about the size and location of lymph nodes, accurately determining whether these nodes are involved in metastatic disease can be difficult.

^18^F-FDG PET/CT in the diagnosis of cervical cancer, preoperative staging, postoperative restaging, efficacy monitoring, and prognosis evaluation plays a more and more important role. Sandro et al. results suggested that combined PET/CT is valuable for preoperative lymph node staging in patients with early cervical cancer, especially lymph nodes with a short axis diameter greater than 0.5 cm (20). In the context of LNM evaluation, the SUVmax serves as an important indicator of nodal involvement. Higher SUVmax values are often associated with a higher likelihood of metastatic spread to lymph nodes (21). Due to its susceptibility to noise and its inability to reflect the overall metabolic level of tumors, the SUVmax has limited value in predicting LNM or prognostic evaluation of cervical cancer (22, 23). Additionally, physiological uptake of adjacent structures such as the ureter and bladder occasionally lead to increased FDG uptake in lymph nodes, or high FDG uptake in reactive hyperplasia lymph nodes, resulting in false positives. However, the relationship between the characteristics of primary tumors and the likelihood of LNM has long been a topic of debate. Therefore, it is of clinical importance to understand whether certain features of primary tumors can be used as predictors of LNM. The objective of our study is to investigate the utility of ^18^F-FDG PET/CT parameters, specifically the SUVpeak, TLG, and MTV of the tumor, in predicting LNM in patients with stage I-II cervical cancer. Currently, the use of ^18^F-FDG PET/CT as a potential marker of tumor viability has gained widespread popularity in the field of oncologic imaging (24–26). Our results showed that the higher SUVmax, SUVmean, SUVpeak, TLG, and MTV of tumor were significantly associated with an increased likelihood of LNM in stage I-II cervical cancer. Suggesting that the potential utility of ^18^F-FDG PET/CT semi-quantitative parameters, especially the TLG, derived from the primary tumor in predicting LNM in stage I-II cervical cancer. TLG is a comprehensive semi-quantitative parameter that reflects the metabolic activity of the whole tumor (21, 27). Compared with SUVmax, TLG could assess tumor burden more accurately. A study involving patients with early cervical cancer showed that tumor MTV and TLG values are important imaging indicators for predicting lymphatic metastasis, especially in patients with PET negative lymph nodes (27).

SCC-Ag is a specific biomarker that is produced by squamous cell carcinoma cells. It serves as a useful tool for diagnosing and monitoring squamous cell carcinoma (16). Elevated levels of SCC-Ag may indicate the presence of squamous cell carcinoma or the progression of the disease. Recent studies have provided evidence linking preoperative serum SCC-Ag levels in patients with cervical squamous cell carcinoma to important clinicopathological factors, including tumor size and LNM (17, 28). Our current study found that LNM patients showed significantly increased SCC-Ag levels compared with NLNM patients. A previous study has shown that the SCC-Ag may be a useful marker for predicting LNM of cervical cancer, especially in stages IB1 and IIA1, and the combination of SCC-Ag and CT may help identify patients with LNM to provide them with the most appropriate therapeutic approach (16, 17, 29). Lymph node involvement indicates that the cancer has spread beyond the primary site, potentially leading to a poorer prognosis. The link between SCC-Ag levels and LNM highlights the potential utility of SCC-Ag as a prognostic marker for assessing disease progression and risk stratification (16). However, the predictive value of SCC-Ag in determining LNM remains a subject of controversy and debate. A study using similar methods suggests that a combination of SCC-Ag level, SUVmax, and TLG is an important prognostic indicator for cervical cancer (16). Therefore, combined factors should be suggested to better reflect the value of ^18^F-FDG PET/CT and SCC-Ag in predicting LNM in stage I-II cervical cancer.

To comprehensively evaluate LNM in stage I-II cervical cancer, our study employed not only ^18^F-FDG PET/CT semi-quantitative parameters and SCC-Ag findings but also conducted univariate and multivariate logistic regression analysis to identify additional predictive factors. The result was that the TLG of tumor remained an independent predictor of LNM in stage I-II cervical cancer, and the cut-off value was 72.18. Multivariate analysis showed that SUVmax and TLG were independent predictors of positive and moderate risk status (21). In high-risk cervical cancer, MTV and TLG were associated with pelvic lymph node metastasis and parametria involvement (21). Different from the results of our study, the reason may be that the TLG is a comprehensive parameter that reflects tumor metabolic activity based on MTV. For further research, we evaluated the predictive LNM models in stage I-II cervical cancer. Our study showed that Model 3, combining the SUVpeak of the tumor, SCC-Ag and TLG of the tumor, had a greater AUC than Model 1and Model 2. Additionally, we observed that the TLG of tumor offered independent and additional information in predicting LNM. Our study revealed significant associations between preoperative levels of SCC-Ag, SUVpeak, and TLG of tumor with the response of LNM in patients with cervical cancer. Incorporating these biomarkers and ^18^F-FDG PET/CT parameters into clinical practice could aid in risk stratification and guide treatment decisions for improved patient outcomes.

There are some limitations in our study. Firstly, it was a retrospective single center study, which means that the data were collected from a single institution and may not represent the broader population. Our study was conducted on a relatively small sample size, which may limit the generalizability of our findings. Secondly, the impact of the partial volume effect on quantitative accuracy in ^18^F-FDG PET/CT imaging, specifically in relation to bladder imaging agent retention. Thirdly, our study only focuses on the predictability of ^18^F-FDG PET/CT semi-quantitative parameters of tumor combined with SCC-Ag for LNM in stage I-II cervical cancer, and further analyze the significance of lymph node metabolic parameters in cervical cancer lymph node metastasis.

## Conclusion

The combination of ^18^F-FDG PET/CT semi-quantitative parameters and SCC-Ag has proven to be a reliable and feasible technique for predicting LNM in stage I-II cervical cancer. The TLG of tumor has been identified as an independent factor for patients with LNM in stage I-II cervical cancer.

## Data availability statement

The original contributions presented in the study are included in the article/supplementary material. Further inquiries can be directed to the corresponding authors.

## Ethics statement

The studies involving human participants were reviewed and approved by Ethical Committee of Hunan Cancer Hospital. The studies were conducted in accordance with the local legislation and institutional requirements. The participants provided their written informed consent to participate in this study. Written informed consent was obtained from the individual(s) for the publication of any potentially identifiable images or data included in this article.

## Author contributions

C-ZJ: Writing – review & editing, Writing – original draft, Methodology, Formal Analysis, Data curation, Conceptualization. KZ: Writing – original draft, Formal Analysis, Data curation. Y-YZ: Writing – original draft, Formal Analysis, Data curation. JY: Writing – review & editing. HY: Writing – review & editing, Writing – original draft, Methodology, Formal Analysis, Conceptualization. XP: Writing – review & editing, Writing – original draft, Methodology, Formal Analysis, Data curation, Conceptualization.
